# Alginate Cryogels as a Template for the Preparation of Edible Oleogels

**DOI:** 10.3390/foods13091297

**Published:** 2024-04-23

**Authors:** Sladjana Meseldzija, Jovana Ruzic, Jelena Spasojevic, Milan Momcilovic, Arash Moeini, Gustavo Cabrera-Barjas, Aleksandra Nesic

**Affiliations:** 1 Vinca Institute of Nuclear Sciences, National Institute of the Republic of Serbia, University of Belgrade, Mike Petrovica–Alasa 12–14, 11 000 Belgrade, Serbia; sladja_ms@vin.bg.ac.rs (S.M.); jruzic@vin.bg.ac.rs (J.R.); jelenas@vin.bg.ac.rs (J.S.); 2 Faculty of Sciences and Mathematics, University of Nis, Visegradska 33, 18 000 Nis, Serbia; milanmomcilovic@yahoo.com; 3 TUM School of Life Sciences, Technical University of Munich, D-85354 Freising, Germany; arash.moeini@tum.de; 4 Facultad de Ciencias para el Cuidado de la Salud, Universidad San Sebastian, Campus Las Tres Pascualas, Lientur 1457, Concepción 4080871, Chile; gcabrerab@gmail.com

**Keywords:** oleogels, alginate, freeze-drying, edible oils, functional food

## Abstract

A high consumption of solid fats is linked to increased inflammation and a risk of cardiovascular diseases. Hence, in recent years, there has been increasing interest in the development of oleogels as a fat substitute in food products. Oleogels are edible gels that contain a large amount of liquid oils entrapped in a 3D network and that can potentially be applied to spreads, bakery goods, meat, and dairy products in order to lower their saturated fat content while maintaining a desirable food texture and mouthfeel. In this work, alginate cryogels were studied as templates for three different edible oils in the process of oleogel formation. Two different freezing regimes to obtain cryogels were employed in order to evaluate better the textural and morphological capabilities of cryogels to adsorb and retain edible oils. It was shown that rapid freezing in liquid nitrogen produces alginate cryogels with a lower density, higher porosity, and a greater ability to adsorb the tested oils. The highest uptake and holding oil capacity was achieved for olive oil, which reached a value of 792% and 82%, respectively. The best chewiness was found for an oleogel containing olive oil, whereas oleogels with the other two tested oils showed better springiness. Hence, the results presented in this work demonstrated that alginate-based cryogels can be effectively used as templates for oleogels and potentially find applications in the food industry.

## 1. Introduction

Fats represent an essential part of a healthy diet; however, their excessive intake, particularly saturated and trans types of fats, can lead to several health problems. A high consumption of saturated and trans fats can contribute to an increased risk of heart diseases [1,2]. Namely, these fats can clog arteries and increase blood pressure, putting strain on the heart. Moreover, excess fat, regardless of the type, can contribute to weight gain and obesity, a risk factor for diabetes, certain cancers, and joint problems [3,4]. One innovative solution that is still in the development stage is the use of oleogels with a profile of healthier fats or with a more controlled uptake of saturated/trans fats. One of the key benefits of oleogels is their versatility. They can be formulated with various oils, allowing for tailoring their fatty acid profile to suit specific nutritional needs or desired tastes. Moreover, oleogels can be designed to have specific melting points and textures, making them ideal for a wide range of culinary applications [5]. They can be used as additives to spreads, healthier alternatives in baked goods, and even to support the creation of structured fillings and toppings with unique functionalities [6,7].

The most common methods of preparing oleogels are the emulsion-templated approach and the foam-templated approach [8,9,10]. In the first case, oil-in-water emulsion is prepared through the use of emulsifiers and stabilizers and additionally dried in an oven or by freeze-drying. One major challenge with emulsion oleogels is the potential for the oil droplets to coalesce and separate over time, impacting the texture and shelf life of the product. The foam-templated approach considers first the preparation of hydrogels or foams and their subsequent freeze-drying in order to obtain a macroporous three-dimensional network. Once the stable cryogel is formed, it is used as a template to adsorb the oil in its voids. Compared to the emulsion-templated method, production via the foam-templated method requires fewer additives, and its can offer inherent stability due to the absence of dispersed droplets and the presence of a continuous gel network [11,12].

Alginate is a naturally occurring polysaccharide found within the cell walls of brown seaweed and holds immense potential in various fields due to its unique properties and versatility [13]. This biocompatible and readily available biopolymer has the ability to form gels in an acidic environment and in the presence of divalent cations. Due to its gelling ability, it has been widely used in the food sector as a thickening and gelling agent [14,15]. In fact, alginate was approved by the Food and Drug Administration (FDA) as a GRAS chemical in food, pharma, and medicine (wound, bone) applications in 1970 [16]. Beyond its gelling properties, alginate possesses several other desirable features. It is non-toxic, biodegradable, and exhibits excellent biocompatibility, making it a safe and sustainable option for diverse applications. This combination of properties paves the way for alginate’s utilization in various fields, including food science, medicine, drug delivery, and environmental engineering [17,18,19,20].

To date, proteins and polysaccharides, separately or in combination, have been investigated for the production of oleogels [21]. To the best of our knowledge, this is the first report related to using alginate cryogels as a template for edible oils in the process of oleogel production. The aim of this work is to develop an alginate cryogel template for three different edible oils. The influence of two different freeze-drying regimes on the textural and morphological properties of the obtained cryogels was studied. In addition, the adsorption and holding capacity of corn, olive, and sunflower oil were presented, as well as detailed mechanical profiles of oleogels, including their compressive strength, Young’s modulus, chewiness, cohesiveness, and springiness. The present work could help to further unlock the potential of alginate cryogels and oleogels in the food sector as saturated/trans fat replacements in food products.

## 2. Materials and Methods

### 2.1. Chemicals

Alginic acid sodium salt was purchased from Acros Organics (Geel, Belgium). CaCl_2_ × 2H_2_O was purchased from Sigma Aldrich (St. Louis, MO, USA). Corn oil (CO), olive oil (OO), and sunflower oil (SO) of high purity were purchased from a local market, Belgrade, Serbia. The contents of different types of fats in the tested oils according to the product labels are presented in Table 1.

### 2.2. Preparation of Cryogels

Alginate cryogels were prepared according to the protocol reported by Rodriguez-Dorado et al., with slight modifications [22]. Alginate was dissolved in water to obtain a 3 wt% solution. The gelation reaction was induced in the presence of a calcium chloride solution. The molar ratio of alginate to Ca^2+^ ions was 2:1. The crosslinking reaction occurred through the slow diffusion of the calcium chloride solution into an alginate solution. The mixture was left until complete gelation, which took place after 12 h. Prior to freeze-drying, the obtained hydrogels were frozen directly through two freezing methods: in a freezer at −20 °C or by immersion in liquid nitrogen (−196 °C). The freezing of hydrogels at −20 °C took place for several hours, whereas freezing in liquid nitrogen was achieved in 10 s. The frozen samples were freeze-dried at −60 °C and a vacuum pressure of 0.011 mbar for 24 h using an Alpha 1–2 Ldplus Freeze-Drier (Martin Christ Gefriertrocknungsanlagen GmbH, Harz, Germany). The following labels were used: AFR for the samples frozen in a freezer at −20 °C and ALN for the samples frozen in liquid nitrogen. Oleogels were obtained through the immersion of AFR and ALN cryogels in three different oils: corn oil, olive oil, and sunflower oil. The codes for these samples are AFR-CO (or ALN-OO), AFR-OO (or ALN-OO), and AFR-SO (or ALN-SO), respectively.

### 2.3. Characterization

#### 2.3.1. SEM

The surface morphology of the samples was monitored with a Hitachi S-4800 scanning electron microscope (SEM) (Hitachi High-tech, Tokyo, Japan) with an accelerating voltage in the range between 5 and 10 kV.

#### 2.3.2. Porosity and Brunauer–Emmett–Teller Surface Area Analysis (BET)

The bulk density of the samples was calculated as the mass-to-volume ratio, and the presented results are an average of three measurements. The porosity of the cryogels was evaluated by the method reported by Onwukamike et al. [23], according to the following equation:

Porosity = (1 − ρ_A_/ρ_R_) × 100, (1)

where ρ_A_ is the bulk density and ρ_R_ is the real density calculated based on the mass fraction of alginate density (1.59 g/cm^3^). All measurements were performed in triplicate and presented as a mean value. The standard deviation was up to 10%.

The BET surface was measured by the nitrogen adsorption-desorption test which was performed by the means of Nova 3000e surface area analyzer (Quantachrome Instruments, Bointon Beach, FL, USA).

#### 2.3.3. Oil Uptake and Holding Capacity

In order to obtain oleogels, the cryogels were accurately weighed (m_1_, g) and then immersed in three different edible oils: corn oil, olive oil, and sunflower oil. The uptake of oils (OU, %) by alginate cryogels was measured by Li et al.’s method, as described in the study [24]. Once the cryogels were immersed in oils, the equilibrium time of the oil uptake process was monitored. Once the sorption process was complete, the formed oleogels were taken out, and the excess surface oil was removed using filter paper. Afterwards, the oleogels were weighed (m_2_, g). The oil uptake of the cryogel templates was measured according to the following equation:

(2) $$OU\left(\%\right)=\frac{m_{2}-m_{1}}{m_{1}}\times 100$$

The oil-holding capacity (OHC, %) was measured according to the method described by Xie et al. [25] and Genc et al. [26]. The obtained oleogels were placed in centrifuge tubes and then centrifuged at 10,000 rpm for 15 min in order to estimate the oil-holding capacity of the cryogels. The oil-holding capacity was calculated according to the following equation:

(3) $$OHC,\%=\frac{m_{1}}{m_{2}}\times 100$$

where m_2_ is the weight of the remaining oil in the oleogel after centrifugation and m_1_ is the total weight of oil before centrifugation. All measurements were performed in triplicate and presented as a mean value. The standard deviation was up to 10%.

#### 2.3.4. Mechanical Properties

The measurements of AFR’s and ALN’s textural and mechanical properties were performed by using a testing machine, an Instron dynamometer (model 1185), equipped with a 10 kN load cell, at room temperature and 50 ± 3% relative humidity. The diameter of all the samples was 21 ± 0.5 mm, with a 7.5 ± 0.3 mm height. Prior to the measurements, the samples in oil were drained for 5 min at room temperature. A modified testing protocol was used for texture profile analysis (TPA) [24,27], with a crosshead speed of 0.5 mm/min for loading and 1 mm/min for unloading for both compression cycles. The maximum applied load for the first compression (P1) was set after reaching 30% of the sample height, while the maximum load for the second compression (P2) was chosen to be 80% of P1. This modified testing protocol ensures no plastic deformation of the tested samples and the full recovery of the samples’ shape and size. All tests were performed 5 times for each formulation, and mean values are presented in the results. The standard deviation for all parameters was up to 10%.

**Figure 1:**
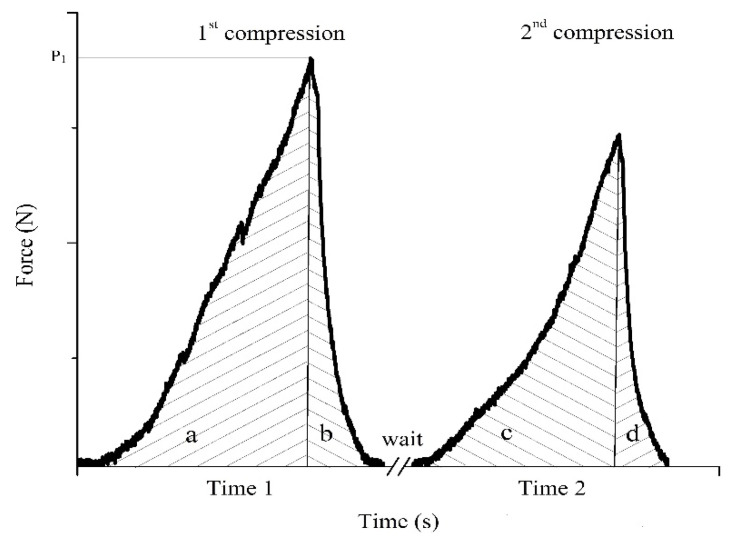
Two compression cycles, where P_1_ is the maximum applied load in the 1st compression. Time 1 is the time needed to reach the maximum load in the 1st compression, and Time 2 is the time taken to reach the maximum load in the 2nd compression.

Texture properties were calculated using the following relations through the extraction of data from Figure 1:

Compressive strength (σ, Pa) is the ability of a material to withstand an applied load, i.e., resistance to deformation under the load. It is a bulk property of the material and refers to material stiffness and elasticity, and it is determined by the relation $\sigma =\frac{P_{1}}{A}$, where P_1_ (N) is the maximum load reached during the first compression and A is the sample’s surface area (mm^2^). It is important to underline that TPA tests in general consider the maximum applied load in the 1st compression, P_1_, as the hardness value of the investigated samples. However, hardness is the material’s resistance to the applied load, but only in a local region, and it is measured as an indenter’s penetration into the material’s surface. Here, we used a compression test to evaluate the strength of the material, considering the whole sample, not just the local region.

The Young’s modulus (E, Pa) depicts how easily a material can be deformed. It is determined by the slope of the linear part of a stress–strain curve.

Springiness (Sp, unitless) presents the viscoelasticity of the material and the time needed for material recovery. It is calculated as $Sp=\frac{Time1}{Time2}$, where Time 1 and Time 2 are the times needed to reach the maximum load in the 1st compression and 2nd compression, respectively.

Cohesiveness (Coh, unitless) describes the consistency of the material, and it is defined by the relation $Coh=\frac{\left(c+d\right)}{\left(a+b\right)},$ where (a + b) is the area under the force–time curve of the 1st compression and (c + d) is the area under the force–time curve of the 2nd compression. If the value of cohesiveness is closer to zero, the material is prone to completely decomposing, meaning that cohesiveness characterizes the extent to which food material maintains its size and shape between the first and second chews.

Chewiness (Che, N) indicates the force required for chewing a material, i.e., the material’s resistance to compressions produced by the teeth during chewing. It is measured through the relation $Che=Coh\cdot Sp\cdot P_{1}$.

#### 2.3.5. Statistical Analysis

The data obtained for the density, porosity, oil uptake and holding capacity, and mechanical tests were analyzed using the SPSS^®^ v.11.0 software (IBM, Chicago, IL, USA), using a two-way analysis of variance (ANOVA) and Tukey’s test (α = 0.05) for mean comparison.

## 3. Results and Discussions

### 3.1. Morphology of Cryogels

**Figure 2:**
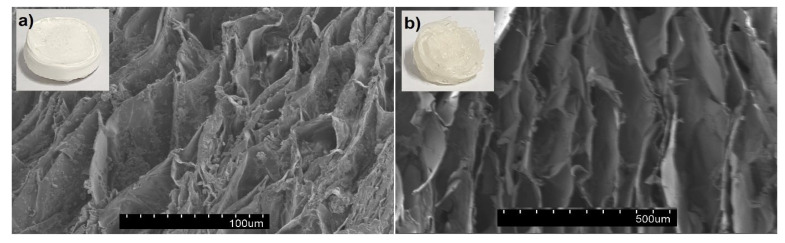
SEM micrographs of (**a**) ALN and (**b**) AFR cryogels.

The cryogel samples slowly frozen at −20 °C (AFR) had a friable physical appearance with an irregular shape in comparison to the samples frozen in liquid nitrogen (ALN). The SEM analysis of the AFR and ALN cryogels revealed structures of weakly bound sheets (Figure 2). The rapid freezing of a sample (ALN) caused a decrease in the size of voids in the cryogel network, essentially because the water crystallization effect was minimized. The void size ranged between 30 and 200 µm. In addition, partially porous structures on the walls of the sheet layers of the ALN sample were detected. On the other hand, the AFR sample had a more irregular and disconnected morphology, with a void size ranging between 200 and 800 µm. Sheet-like structures are generally related to the slow growth of water crystals during freezing and drying processes, which leads to a compression of the gel network into less porous planar aggregates. The slow freezing of hydrogels can further accelerate irregular crystal growth, leading to larger voids with a non-homogenous size distribution in the network. Similar morphological structures are observed for the freeze-dried pectin [28,29], alginate [30], and starch cryogels [31].

### 3.2. Porosity and BET

The bulk density, porosity, and surface area are crucial properties in assessing cryogels due to their significant impact on the performance and functionality of these materials. The bulk density of the ALN sample was 0.06 g/cm^3^, and for the AFR sample, it was 0.1 g/cm^3^. The lower bulk density of the cryogels that were frozen in liquid nitrogen compared to those frozen in a standard freezer was due to the difference in freezing rates. In general, when a cryogel comes into contact with liquid nitrogen, it experiences a very rapid and uniform freezing process. This rapid freezing does not allow for the formation of large ice crystals within the material. With a smaller formation of ice crystals, the spaces left behind after the ice sublimates during the drying phase are also smaller, thus resulting in an interconnected network of pores within the cryogel, leading to a lower overall density. On the other hand, freezers typically operate at significantly higher temperatures (around −20 °C) than liquid nitrogen (−196 °C), which leads to a slower freezing process, providing more time for the formation of larger ice crystals. The formation of larger ice crystals in the freezer leads to larger voids being left behind after sublimation, thus creating a cryogel with a more open and coarse pore structure, ultimately leading to a higher bulk density compared to its liquid-nitrogen-frozen counterpart. The same pattern in the bulk density of starch cryogels frozen in liquid nitrogen and a in freezer is obtained in the literature [31]. The density values obtained for the alginate cryogels were similar to those for chitosan cryogels reported in the literature [32,33]. The liquid displacement method was utilized to estimate the porosity of the cryogels. Ethanol was used as a displacement liquid since it can easily penetrate through pores, but it does not influence a change in the volume of the tested cryogels. The estimated porosity for the ALN and AFR samples was 75% and 10%, respectively. These results are expected since density is inversely proportional to the porosity parameter of materials. Nitrogen adsorption was used to measure the specific surface area with the Brunauer–Emmett–Teller (BET) isotherm. The maximum measured BET surface area (S_BET_) of a freeze-dried sample was achieved for the ALN sample (18.7 m^2^/g), which was almost four times higher than for the AFR sample (4.5 m^2^/g). The differences in the S_BET_ values could be explained by the different pore size distributions of the cryogels and their morphology. It should be noted that large macropores cannot be detected by a BET analysis, thus preventing a complete pore size distribution evaluation based solely on the BET results. However, the SEM analysis revealed that the ALN cryogels had a lower pore size than the AFR cryogels. The pore size range combined with the porosity values of the cryogels indicated that the ALN sample had more interconnected pores with smaller sizes in comparison to the AFR sample, with fewer and more disconnected pores of larger sizes. This architecture of the cryogels led to a higher S_BET_ value for the ALN sample. Overall, it can be concluded that the freezing regime significantly influenced the architecture and density of the alginate cryogels. Similar findings are reported in the literature, where Jimenéz-Saelices et al. obtained a lower *S*_BET_ for nanofibrillated cellulose cryogels with a slow freezing process in comparison to a quick freezing process [34,35].

### 3.3. Oil Uptake and Holding Capacity

**Figure 3:**
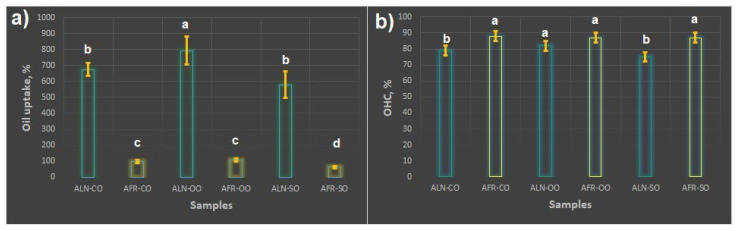
(**a**) Oil uptake and (**b**) holding capacity for ALN and AFR samples. Different letters indicate significant differences, considering *p* < 0.05, according to Tukey’s test.

The oil uptake and oil-holding capacity of the alginate cryogels are presented in Figure 3. Three different oils were used as test models, corn oil, olive oil, and sunflower oil, because they are the most widely used oils in the preparation of food and have good nutritional value. It is important to highlight that the time required to reach sorption equilibrium for all the tested oils in the ALN sample was up to 30 min, whereas for the AFR sample, it was 24 h, indicating that the rate of oil uptake is greatly affected by the density and architecture of cryogels. These differences in oil sorption kinetics could be attributed to the different morphologies of the cryogels. In particular, the ALN cryogel had much smaller and more interconnected pores as compared to the AFR cryogels but a higher S_BET_ value and porosity, which allowed for the faster diffusion of oil molecules inside the cryogel. The AFR sample had fewer pores of larger sizes that were less connected, which contributed to its lower S_BET_ value, thus preventing the easy and fast movement of oil molecules through the cryogel.

Moreover, the ALN sample demonstrated a higher percentage uptake of all the tested oils (ranging between 580 and 792%) than the AFR sample (ranging between 65 and 110%). These results collaborated well with the textural and morphological properties of the cryogels presented in Section 3.1 and Section 3.2. The ALN sample had a lower density, fewer structural collapses, and a higher porosity, which allowed for the faster diffusion of oils through the pores. Contrary to this, the AFR sample demonstrated low porosity with a deformed structure and had pores that were not easily accessible for the fast and high percentage entrapment of oils. It has been previously reported that the uptake of different liquids by porous materials is influenced by different factors such as the pore size, porosity, tortuosity of pores, and internal surface [36,37]. Regarding the uptake of different oils, both samples, ALN and AFR, had the highest uptake for olive oil and the lowest uptake for sunflower oil. This outcome was expected since olive oil, among all the tested oils, has the highest content of monounsaturated fat (oleic acid), which easily penetrates through cryogels, whereas sunflower oil has the highest content of polymonosaturated fats (α-linolenic and linoleic acid), thus impeding the oils’ uptake due to the steric hindrance effect.

Oleogel’s capability to hold oil is one of the important factors in evaluating the capability of foam-templated oleogels in the food industry. The higher the OHC values, the more functional the oleogels are. In this study, the tested oleogels had an OHC ranging between 75 and 87%. Among the ALN samples, the highest OHC value was obtained for olive oil (82%) and the lowest for sunflower oil (75%), thus indicating that the ALN oleogels with a higher load of oil were able to retain the highest amount of oil inside their network. It is interesting to note that AFR samples had higher OHC values despite a lower percentage uptake of oils (around 85–87%) than the ALN samples. In the case of the ALN samples, oil was inserted into the large and small voids inside the cryogel, which could cause more disruption to its internal network than if the oil was partially filled in the AFR cryogels and only in voids that were not easily accessible. This weakening of the network could lead to a less robust structure that might be less effective at retaining oil, particularly under external pressure or flow. Regarding the AFR samples, there were no significant differences (*p* > 0.05) in the OHC values for the three different oils tested. These results suggest that oils are just physically entrapped in the cavities of cryogels and that oils can easily be released by centrifugation. The same trend was obtained by Manzocco et al., where carrageenan aerogels with a higher percentage uptake of sunflower oil (450%) demonstrated the lowest oil-holding capacity (62%), and the sample with the lowest uptake of sunflower oil (250%) had the highest percentage retention of oil inside the network (82%) [36]. The OHC values obtained in this work for alginate-based cryogels are higher than for other biobased oleogels without shearing reported in the literature. For example, Chen and Zhang demonstrated that alginate/soy protein aerogels could absorb corn oil up to 1089% and hold 40% of it [38]. Parisa et al. developed xanthan cryogels with the ability to hold 60% of sunflower oil [39].

### 3.4. Mechanical Analysis

**Figure 4:**
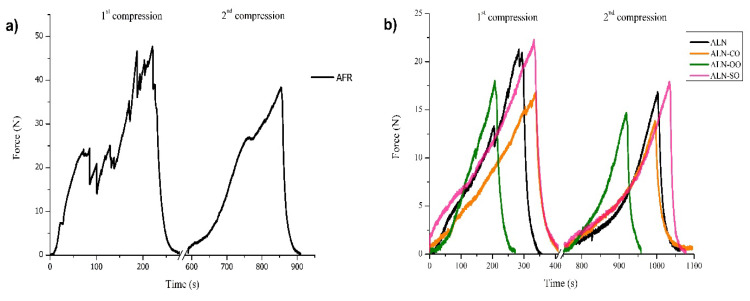
Two compression cycles for (**a**) AFR and (**b**) ALN cryogels and ALN oleogels.

The mechanical characteristics of oleogels are important for evaluating their applicability in the food industry, particularly in the processing and storage of food. Mechanical parameters, in terms of the compressive strength, Young’s modulus, chewiness, cohesiveness, and springiness of alginate cryogels and oleogels, are evaluated and summarized in Table 2, whereas the force–time curves of the tested samples are presented in Figure 4. The compression strength parameter can be associated with the first bite feeling, so it is important from a consumer’s point of view. The highest compressive strength and Young’s modulus were obtained for the AFR sample, which indicated the high strength and stiffness of the material. Moreover, this sample demonstrated a high level of brittleness during the compression test since it started to break, which is evidenced in Figure 4, as evidenced by the presence of many peaks indicating discontinuities in force during loading in first compression. Hence, the AFR-based oleogels were not subjected to further mechanical tests due to their low applicability in real systems. On the other hand, the ALN sample demonstrated a moderate compressive strength of 64.7 kPa and a Young’s modulus of 25.6 kPa. The compressive strength and Young’s modulus of the ALN sample are comparable to data from the literature for alginate [40] and alginate/lignin cryogels [30]. The compressive strength of all the tested oleogels decreased in comparison to the reference ALN cryogel due to the weakened interconnected network upon oil sorption and the reduced contribution of pores to the load distribution. However, although the compressive strength of the ALN-SO sample was slightly lower than that of the ALN sample, there was no significant difference (*p* > 0.05) between these two values. The studies reported in the literature deal only with the evaluation of the firmness/hardness of oleogels in local regions but not with their strength throughout their entire volume; hence, it is not possible to compare the values obtained in this work with those in the literature. Furthermore, the ALN-CO and ALN-SO oleogels had lower Young’s modulus values in comparison to the reference ALN sample, which indicated that the introduction of corn and sunflower oil into the cryogel network reduced the rigidity of the materials. However, in the case of the ALN-OO oleogel, the Young’s modulus was higher than that of the ALN sample, thus showing the stiffness of the material. These differences can be explained by the different contents of monounsaturated and polyunsaturated fats in the oils. Namely, oils with a high content of polyunsaturated fats are, in general, less viscous and induce some sort of plasticization effect in materials, whereas oils with a high content of monounsaturated fats are more viscous and act as a stiffener of materials [41,42,43]. The relationship between the Young’s modulus (stiffness) and compressive strength values obtained in this work can seem counterintuitive at first glance. However, it is important to understand that these are related but not identical properties and that compressive strength values were evaluated only up to 30% cryogel/oleogel compression, not up to the breaking point of the samples. Hence, although the ALN-SO oleogel demonstrated the highest initial compression strength, this does not mean that its maximum compression strength would be higher than that of the other two tested oleogels. On the other hand, the increased stiffness (Young’s modulus) of the ALN-OO sample does not necessarily equate to a stronger material (compressive strength) in all cases. It is suggested that this sample is more brittle despite being stiffer. A stiffer but more brittle material might fracture under a compressive load before reaching its maximum compressive strength.

Chewiness and springiness represent texture parameters that describe the elastic behavior of food. Food is considered elastic if it can recover its original shape after a deformation caused by an applied mechanical force. For example, beef and squid are highly elastic foods [44]. More specifically, chewiness represents the energy required to break solid food by chewing. The chewiness for all tested oleogels was lower than for the ALN cryogel, meaning that less energy should be required to destroy samples and to be able to swallow food products. The values of chewiness obtained in this work correspond well to the chewiness values of cooked meats [45]. The springiness parameter can be described as the bouncing-back ability of foods through consecutive bites, i.e., the rate at which food recovers its shape after the first compression cycle. Low values of springiness indicate a brittle material [46]. The lowest chewiness and springiness values were obtained for the ALN-OO sample, meaning that this oleogel was more brittle than the other samples and a low amount of energy would be required to chew it. The ALN-CO and ALN-SO samples had higher springiness and chewiness values, meaning more mastication energy in the mouth would be required for the food product prior to swallowing. These results are in agreement with the compressive strength–Young’s modulus correlations among the different oleogels. In fact, the ALN-OO oleogel was the stiffest material, but with a lower compression strength than the ALN-SO sample, which indicated its brittle nature, thus requiring less energy for chewing. The ALN-based oleogels containing a high content of polyunsaturated fat (ALN-SO and ALN-CO) were more elastic (had lower Young’s modulus values), thus imparting more gumminess behavior, meaning that more energy was needed to chew the material. Moreover, elastic materials can undergo a larger deformation under stress compared to stiffer materials. This larger deformation allowed for a more noticeable “bounce back” when the stress was released, thus contributing to the higher springiness value in comparison to the ALN-OO sample. The obtained chewiness and springiness values for the ALN-based oleogels are similar to the ones reported in the literature for hydroxypropyl methyl cellulose oleogels [47] and polyglycerol stearate oleogels [48], respectively.

Cohesiveness represents the strength of the internal bonds of a material and its ability to deform before rupturing under an applied mechanical force. A high cohesiveness value is desirable because it means that during the mastication process, food turns into a bolus rather than disintegrating [49]. The lowest cohesive value was obtained for the AFR sample, which was expected since it was already confirmed that this sample did not have a regular and homogenous morphology and was easily plastically deformed. Regarding the ALN cryogel and oleogels, there were no significant differences among the cohesiveness values (*p* < 0.05), and they were close to one, suggesting that these samples did not disaggregate during the test and had some elasticity [50]. Similar cohesiveness values are obtained in the literature for gelatin gels and sausages [51,52].

## 4. Conclusions

In this study, alginate cryogels were investigated as a template for corn, olive, and sunflower oil. In the process of preparing the cryogels, two different freezing regimes were used. It was shown that rapid freezing prior to drying the material induced cryogels with a better morphological structure and higher porosity in comparison to the slow freezing regime. The uptake of the tested oils was in the range of 580 and 792% for the rapidly frozen cryogels, whereas the oil-holding capacity ranged between 67 and 79%. The addition of oils caused a decrease in the compression strength, chewiness, and springiness values, whereas there were no significant differences in the cohesiveness values among the different samples. The results obtained in this work showed that alginate cryogels can be efficient templates for edible oils to process functional oleogels for the food industry.

## Figures and Tables

**Table 1:** Contents of fats in corn, olive, and sunflower oil according to the labels on the products.

Oil	Saturated Fat %	Monounsaturated Fat %	Polyunsaturated Fat %
Corn oil	13	28	55
Olive oil	13	70	9
Sunflower oil	12	23	65

**Table 2:** Mechanical parameters of alginate-based cryogels and oleogels.

Sample	Compressive Strength * (KPa)	Young’s Modulus (KPa)	Chewiness (N)	Springiness	Cohesiveness
AFR	178	79.0	63.8	1.79	0.748
ALN	64.7	25.6	39.4	2.28	0.812
ALN-CO	48.9	18.0	28.8	2.17	0.793
ALN-OO	56.7	33.6	24.1	1.68	0.797
ALN-SO	64.1	20.0	37.6	2.09	0.808

* Calculated using maximum applied force at 30% of the sample height.

## Data Availability

Data is unavailable due to privacy and ethical restrictions. The data presented in this study are available on request from the corresponding author.
